# Low physical activity is a risk factor for sarcopenia: a cross-sectional analysis of two exercise trials on community-dwelling older adults

**DOI:** 10.1186/s12877-024-04764-1

**Published:** 2024-02-29

**Authors:** Onni Hämäläinen, Anna Tirkkonen, Tiina Savikangas, Markku Alén, Sarianna Sipilä, Arto Hautala

**Affiliations:** 1 Faculty of Sport and Health Sciences, University of Jyväskylä, Jyväskylä, Finland; 2 Gerontology Research Center, University of Jyväskylä, Jyväskylä, Finland; 3 Department of Medical Rehabilitation, Oulu University Hospital, Oulu, Finland

**Keywords:** EWGSOP2, Insufficiently physically active, Hip fracture, Prevalence

## Abstract

**Background:**

Physical inactivity is an important factor in the development of sarcopenia. This cross-sectional study explores the prevalence of sarcopenia and associations of physical activity (PA) with sarcopenia in two exercise trial populations. These study groups are clinically meaningful community-dwelling populations at increased risk for sarcopenia: older adults not meeting the PA guidelines and those with a recent hip fracture (HF).

**Methods:**

Data from 313 older adults who did not meet the PA guidelines (60% women; age 74.5 ± 3.8, body mass index 27.9 ± 4.7) and 77 individuals with HF diagnosed on average 70 ± 28 days earlier (75% women; age 79.3 ± 7.1, body mass index 25.3 ± 3.6) were included in this study. Grip strength and muscle mass (Dual-energy X-ray absorptiometry [DXA] in older adults not meeting the PA guidelines and bioimpedance analysis in participants with HF) were used to assess sarcopenia according to the European Working Group in Older People 2019 (EWGSOP2) criteria. The current level of PA was self-reported using a question with seven response options in both study groups and was measured with a hip-worn accelerometer for seven consecutive days in older adults not meeting the PA guidelines.

**Results:**

The prevalence of sarcopenia and probable sarcopenia was 3% (*n* = 8) and 13% (*n* = 41) in the older adults not meeting the PA guidelines and 3% (*n* = 2) and 40% (*n* = 31) in the HF group, respectively. In the age- and sex-adjusted logistic regression model, the lowest levels of self-reported PA were associated with increased probable sarcopenia and sarcopenia risk in older adults not meeting the PA guidelines (OR 2.8, 95% CI, 1.3–6.1, *p* = 0.009) and in the HF group (OR 3.9, 95% CI, 1.4–11.3, *p* = 0.012). No significant associations between accelerometer-measured PA and probable sarcopenia or sarcopenia were found.

**Conclusions:**

Probable sarcopenia is common among community-dwelling older adults not meeting the PA guidelines and very common among individuals recovering from HF who are able to be involved in exercise interventions. In addition, since low PA is associated with higher probable sarcopenia and sarcopenia risk, it is recommended to screen for sarcopenia and promote regular physical activity to prevent sarcopenia in these populations.

**Supplementary Information:**

The online version contains supplementary material available at 10.1186/s12877-024-04764-1.

## Background

Sarcopenia is a condition characterized by the occurrence of progressive and general loss of muscle strength and muscle mass [1]. Sarcopenia is associated with an increased risk of adverse health outcomes [2], such as functional decline, hospitalization, mortality [3], and increased health care costs [4–6]. Furthermore, the predicted aging of the population [7] and increase in the prevalence of sarcopenia [8] make it a clinically and globally important geriatric syndrome. In 2019, the European Working Group on Sarcopenia in Older People updated the consensus definition and diagnosis for sarcopenia (EWGSOP2). The proposed consensus diagnosis for sarcopenia includes both low muscle strength and low muscle mass, while probable sarcopenia is defined as having low muscle strength only. In addition, severe sarcopenia can be diagnosed if a person has low physical performance, usually measured as low habitual walking speed, in addition to low muscle strength and mass [9]. A recent meta-analysis estimated that the global prevalence of sarcopenia in individuals 60 years old and older is 10–27% depending on the population and definitions used [10]. In another meta-analysis, the lowest prevalence of sarcopenia was seen in community-dwelling women (9%) and men (11%), while the highest prevalence was seen in men living in nursing homes (51%) [11].

Age-related lifestyle factors, such as physical inactivity and inadequate nutrient intake, several diseases, hormonal factors, and chronic inflammation, are known to affect the pathways responsible for protein synthesis and proteolysis [12]. Eventually, changes in these pathways with other physiological mechanisms during aging [12–14] result in an imbalance in protein metabolism and loss of muscle mass and strength in older adults. One important factor to consider in the development of sarcopenia is myosteatosis, in which intermuscular adipose tissue has been found to accumulate with age and can lead to metabolic and muscle dysfunctions and poor muscle quality [15]. Recently discussed and still evolving concepts have identified sarcopenia as acute or chronic [9, 16]. This concept recognizes acute illness or injuries (e.g., HF and hospital admission) as the main reasons for acute sarcopenia [9], while aging itself, sedentary lifestyle, muscle disuse, inadequate nutrient intake, and chronic diseases could be the main reasons of slowly developing sarcopenia [1].

Physically inactive older adults are in a major risk for slowly developing sarcopenia. Meta-analyses performed in 2017 and 2021 showed that older adults with low or no regular PA were at a 1.7- to 2-fold greater risk of having sarcopenia compared to physically active individuals [17, 18]. In another study with 2309 older adults, those with lowest baseline levels of self-reported moderate to vigorous PA (MVPA) had a 1.5-fold greater incidence of sarcopenia over a 5-year period compared to those with moderate to high levels of self-reported MVPA [19]. In European countries, higher proportion of physically active subjects (fulfilling the PA guidelines) represented lower proportion of prefrailty or frailty, and vice versa [20]. According to population survey in Finnish over 70 years old adults, only 16% met the PA recommendations for both, aerobic and strength exercise, and 35% met the recommendations for aerobic exercise [21]. In this study, we aimed to identify if the level of PA may influence on sarcopenia risk in those individuals who are not meeting the PA recommendations but are motivated and capable to participate in exercise intervention. We feel this is important in aiming to identify those community-dwelling older individuals who might best benefit from preventive measures of sarcopenia risk.

An important risk group for acute sarcopenia are those with a recent HF. HF is a severe insult, that causes acute inflammation stress [22], and fracture-related hospital admission including bedrest and muscle disuse, that all can accelerate the loss of muscle mass and strength in older adults [23–25]. Park et al. (2022) found, that in older adults with low-energy HF, muscle mass declined about 0.5 kg/m^2^, and the prevalence of sarcopenia increased up to 57% and 89% in men and women, respectively, after 1- to 2-years following HF [26]. Additionally, a higher prevalence of sarcopenia has been reported in participants with a hip fracture than in those with no hip fracture [27, 28].

After HF some individuals get institutionalized, and from those who discharged to home many experience mobility difficulties and may therefore be unable to perform activities of daily living [29]. It is acknowledged as well, that adequately targeted rehabilitation interventions after HF are important [30]. HF population in this study represents not the frailest, but those discharged individuals who experience either moderate or severe mobility difficulties, but still might have enough capacity to attain exercise-induced mobility benefits [31]. In this study, we aim to understand the associations between PA and sarcopenia, which both are important risks for functional decline [1], and to identify risk groups for further measures to prevent and care sarcopenia with targeted rehabilitation in community-dwelling older adults recovering from HF.

Physical activity and exercise are potential agents to counteract muscle loss and strength decline [32]. As shown in a recent meta-analysis, resistance training increases muscle strength, muscle mass, and physical performance in individuals with presarcopenia, sarcopenia, or frailty [33]. Additionally, higher levels of physical activity has been shown to protect against sarcopenia in observational [17] and longitudinal settings [19].

Although there are many studies on the association between physical activity and sarcopenia risk, latest meta-analysis conducted in 2017 and 2021 [17, 18] and, to our best knowledge, studies published since on the association between PA and sarcopenia have neither examined community-dwelling older adults not meeting PA guidelines nor those with a recent HF. Both groups are in a high risk for developing sarcopenia and related adversities. As insufficient PA is common among older adults [34] and the annual number of HF is expected to grow in the future [35], more information on muscle mass and function is needed to support the promotion of functional capacity in these groups. In this study, we are aiming to understand better associations of physical activity and sarcopenia in these at-risk populations, but focusing on samples that presents not the frailest or the most active ones, and who are capable and motivated to participate in exercise trials.

Therefore, the aims of this secondary cross-sectional analysis study were: (1) determine the prevalence of sarcopenia, and (2) determine the associations between PA levels and sarcopenia classifications according to EWGSOP2 in community-dwelling 70- to 85-year-old men and women not meeting the PA guidelines, and over 60 years old men and women recovering from HF who are participating in exercise trials.

## Methods

### Study participants

This study comprised two community-based cohorts. The Promoting Safe Walking Among Older People (PASSWORD) study was a randomized, controlled trial (RCT, ISRCTN52388040) including 70- to 85-year-old adults (*n* = 314) who self-reported not meeting the physical activity (2007) recommendations [36] (< 150 min per week of moderate to vigorous PA in bouts of > 10 min and no regular muscle strengthening activities) [37]. The Promoting Mobility After Hip Fracture (ProMo) study was an RCT (ISRCTN53680197) including adults older than 60 years old (*n* = 81) with a recent HF. Baseline measurements in the ProMo study were organized on average 70 (± 28) days after the fracture and 42 (± 23) days after discharged to home. At baseline HF patients were interviewed and 70% of all HF patients informed receiving a written home exercise program including typically 5 to 7 exercises for lower limbs. Adherence to doing exercise varied, while 70% exercised every day, 21% on weekly basis and 9% few times month, and of all patients 12 got referral to physiotherapy [38]. The exclusion criteria in both studies were severe chronic conditions that affect cognitive or physical function. In the PASSWORD -study these diseases were cancer requiring treatment in the past year, severe musculoskeletal diseases (e.g. osteoarthritis, osteoporosis with fragility fracture), severe lung, renal, or cardio-vascular disease, and diabetes with insulin medication, severe psychotic disorder, cognitive impairment (Mini Mental State Examination, MMSE < 24) or disease affecting cognition [37]. In the ProMO exclusion criteria were if patients were confined to bed at the time of fracture, suffering severe memory problems (MMSE < 18), alcoholism, severe cardiovascular, pulmonary, or progressive disease (i.e. neoplasm, ALS), para- or tetraplegic or severe depression (Beck Depression Inventory, BDI > 29) [38]. In both studies, the study physician or nurse made the decisions individually for excluding participants. Still, participants with previously listed diseases, that were not affecting to safety participation to exercise were included. In addition, in the PASSWORD study participants had to be able to safely participate in physical exercise and to walk 500 m without assistance from another person or walking aid, while the ProMo study had no such inclusion criteria.

**Fig. 1:**
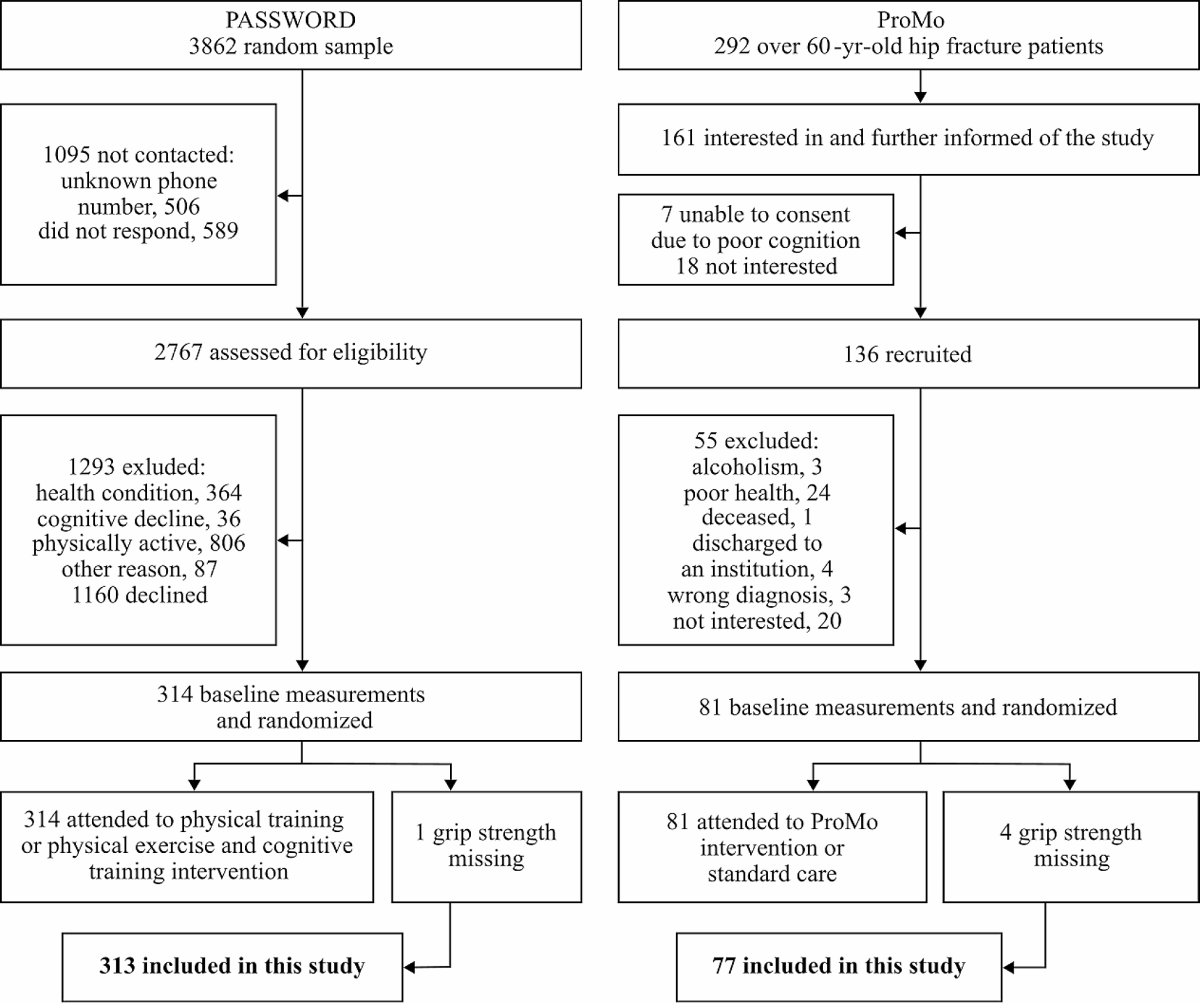
Flow charts of the PASSWORD and ProMo studies and participants included in this study

Participants in the PASSWORD (*n* = 314) and ProMo (*n* = 81) studies who completed baseline measurements for hand grip strength were included in this study (see Fig. 1). Consequently, this study included 313 older adults not meeting the PA guidelines and 77 older adults with recent HF.

All participants were living in Central Finland, and the studies were conducted in the research laboratory of the Faculty of Sport and Health Sciences at the University of Jyväskylä. Participants were given written information about the study, they had an opportunity to discuss it with the researchers before participation, and they signed informed consent forms before baseline measurements. Both studies were approved by the Ethics Committee of Central Finland Health Care District.

### Measurements

#### Sarcopenia

Baseline data from both studies were used. Sarcopenia was assessed using the EWGSOP2 criteria, which consist of grip strength, appendicular skeletal muscle mass and physical performance assessed by the habitual gait speed test [9]. Baseline measurements in the ProMo study were organized on average 70 (± 28) days after the fracture and 42 (± 23, range 4–153) days after discharged to home. Grip strength was measured three times or until no further improvement in strength occurred from the dominant hand using a dynamometer fixed in the arm of a chair (Metitur, Palokka, Finland). The highest value was used in the analysis. The cutoff point for low grip strength was < 16 kg for women and < 27 kg for men [9]. Muscle mass was evaluated with dual-energy X-ray absorptiometry (DXA, Lunar Prodigy, GE Healthcare, Madison, WI, USA) in the PASSWORD study and with a bioimpedance device with eight polar electrodes (BC-418, TANITA, Tokyo, Japan) in the ProMo study. The appendicular skeletal muscle mass index (ASMI) was calculated by dividing appendicular skeletal muscle mass (ASMM) by the square of body height. ASMI values less than 5.5 kg/m^2^ and 7.0 kg/m^2^ were used as cutoff points for low muscle mass in women and men, respectively [9]. Habitual gait speed was assessed in the PASSWORD study over 20 m and in the ProMo study over 10 m. Participants were asked to walk at a self-selected pace along the walkway with pair of light reacting photocells with integrated time measurement system, that measured the walking time [39]. Gait speed was then calculated, and a cutoff value ≤ 0.8 m/s was used when assessing low physical performance for both sexes [9]. As presented in the EWGSOP2 guidelines, the occurrence of probable sarcopenia was defined as having low grip strength only. Sarcopenia was defined as having both low grip strength and ASMI. The occurrence of severe sarcopenia was defined if all three factors -- low grip strength, low ASMI, and low habitual gait speed -- were present. Only one individual in this study had severe sarcopenia; therefore, categories for severe sarcopenia are not presented. In cases in which data on muscle mass were missing (*n* = 5), participants were categorized into no sarcopenia or probable sarcopenia groups by the grip strength value.

### Physical activity

Self-reported PA was assessed in both study groups using a seven-level physical activity scale. Self-reported PA scales have a good predictive validity with respect to risk factors for adverse health outcomes [40]. Seven-level PA scale correlates weakly with accelerometer measured LPA (*r* = 0.105) and with accelerometer measured MVPA (*r* = 0.318), and it has acceptable test-retest reliability [41]. In adults not meeting the PA guidelines following categories were used to assess the current level of self-reported PA: (0) not moving more than is necessary; (1) casual walks and light outdoor recreation 1–2 times/week; (2) casual walks and light outdoor recreation several times a week; (3) 1–2 times/week brisk physical activity; (4) 3–5 times/week a brisk physical activity; (5) fitness exercises several times a week; and (6) competitive sports and regular exercise [42]. Since there were no responses in category 6 and only one in category 5, category 6 was left out from the analyses, and categories 4 and 5 were combined. For additional model for the logistic regression analysis the responses were recoded as inactivity to low PA (categories 0–2) and moderate to vigorous PA (categories 3–5).

In the HF group, the level of self-reported PA for the preceding month was assessed with the following categories: (0) mostly resting or low physical activity; (1) sitting activities; (2) low physical activity; (3) moderate physical activity (MPA) 3 h/week; (4) MPA at least 4 h/week; (5) fitness sports multiple times per week; and (6) competitive sports and regular exercise. Since there were no responses in category 6, it was left out of the analyses. In addition, there was only 1 answer in categories 4 and 5; therefore, categories 4 and 5 were combined with category 3. Due to low response frequencies in the lowest and highest PA categories, additional models for different category combinations (categories 0–1 and 2–5 and categories 0–1, 2, and 3–5) were used in the logistic regression model.

Accelerometry data were only available for adults not meeting the PA guidelines. Physical activity was recorded using a hip-worn tri-axial accelerometer, model UKK RM 42 (UKK Terveyspalvelut, Tampere, Finland), for seven consecutive days. Accelerometer was kept in an elastic waistband above the iliac crest on the right side. Participants were instructed to wear the accelerometer during waking hours, except during water-based activities. Participants kept diary on wearing hours and reasons for taking the accelerometer off. Data from participants with at least three days of wearing time of at least 10 h per day were included in this study analysis. The median resultant acceleration (g) of five-second nonoverlapping epochs was used to calculate daily mean minutes for PA. The level of PA was divided using defined and validated cutoff points for sedentary behavior (SED, bin threshold < 0.0167 g), light physical activity (LPA, ≥ 0.0167 to 0.091 g), moderate activity (≥ 0.091 to < 0.414 g) and vigorous activity (≥ 0.414 g) [43, 44]. Due to the small amount of vigorous-intensity PA, moderate and vigorous PA were combined into moderate to vigorous PA (MVPA). There were 21 participants (17 women, 4 men) missing data on accelerometer-measured PA. The reasons for data missing were insufficient use of accelerometer (*n* = 3) and technical issues (*n* = 18). The accelerometry procedure has been described in detail previously [45].

### Other variables

The measurements of other variables have been described previously in detail [37, 38]. Briefly, for both study groups, physical characteristics included age and body mass index, DXA (PASSWORD) and bioimpedance (ProMo) measured fat percent (FP). Physical performance was assessed with the short physical performance battery (SPPB), including the five times chair stand test, walking test (over 4 m in PASSWORD and over 2.44 m in the ProMo) and static balance tests [46]. Maximal isometric knee extension strength was measured with adjustable dynamometer chair (Metitur Ltd, Palokka, Finland) on the dominant hand side (PASSWORD) and on the fractured and non-fractured side (ProMo). The ankle was attached to a strain-gauge and knee was set at angle of 60º. After familiarization three maximal efforts with 30 s rest were conducted, and the best of the three trials was accepted as the result [47]. During clinical examinations, the study physician checked chronic diseases (musculoskeletal pain/diseases, cardiovascular diseases, pulmonary diseases, osteoarthritis, diabetes, stroke/transient ischemic attack, heart failure/valve diseases, cancers, depression, and other diseases) from the health registry. Smoking status, marital status, and highest education was assessed with a questionnaire. Self-rated health was reported by the question “How would you describe your health” with a scale from very good to very poor and dichotomized to very good/good and average/poor, that are valid and widely used scale [48].

### Statistical analysis

Descriptive statistics are shown as the means and standard deviations (SDs) for continuous variables and frequencies (fr) and percentages (%) for categorical variables. Age-adjusted estimated mean value differences in baseline characteristics according to sarcopenia status were assessed using univariate analysis of variance for continuous variables and the chi-square test for categorized variables for both sexes separately. A test for heterogeneity was used to test the equality of variable variances between sarcopenia groups. If variances in the variables were significantly different, nonparametric analysis was used.

An age- and sex-adjusted logistic regression model was used to examine the associations between PA and sarcopenia categories. Independent measures for both study groups were self-reported PA levels (categories), and in older adults not meeting the PA guidelines, the accelerometer measured PA levels of SED, LPA, and MVPA with a 30-min daily increase. The association with accelerometer-measured PA was analyzed using two models and participants with missing accelerometer data was excluded from the analysis. In the first model each PA levels and their association with sarcopenia were analyzed separately. In the second model all PA levels were analyzed in the same regression model and were adjusted for other PA levels. Different combinations of self-reported PA categories were used in the analysis of both study groups because of the low frequencies of the lowest and highest self-reported PA categories. As the prevalence of sarcopenia was low in both study groups, the sarcopenia categories were divided into two categories for logistic regression: sarcopenia/probable sarcopenia group and non-sarcopenia. The non-sarcopenia group was selected as the reference category. *P*-value < 0.05 was considered statistically significant. All analyses were performed using IBM SPSS Statistics software, version 28.0.

## Results

Participant characteristics and baseline measurements for sarcopenia determinants and PA levels are presented in Table 1. Among older adults not meeting the PA guidelines (*n* = 313, mean age 74.5 ± 3.8; body mass index 27.9 ± 4.7, 60% women), 45% perceived their current health status as good or very good, 70% had more than one chronic disease, and 37% reported having brisk physical activity once or more per week. In the HF group (*n* = 77, mean age 79.5 ± 7.0, body mass index 25.3 ± 3.6, 77% women), 61% of individuals perceived their health as good or very good, 87% had more than one diagnosed chronic disease, and 6 participants (8%) reported having at least 3 h of brisk PA per week.

**Table 1:** Descriptive statistics for older adults who are not meeting the physical activity guidelines and older adults with recent hip fractures

	Not meeting PA guidelines (*n* = 313)		Hip fracture group (*n* = 77)	
	Men (*n* = 125)	Women (*n* = 188)	Men (*n* = 18)	Women (*n* = 59)
Age, years	74.4 ± 3.9	74.5 ± 3.8	79.6 ± 5.8	79.5 ± 7.4
Marital status				
Married/living with a partner	102 (82)	96 (51)	9 (50)	20 (34)
Widowed/separated/not married	23 (18)	92 (49)	9 (50)	39 (66)
Highest education				
Low	27 (22)	21 (11)	9 (50)	40 (68)
Mid-level	78 (62)	122 (65)	3 (17)	13 (22)
High	20 (16)	45 (24)	3 (17)	6 (10)
Body mass index, kg/m^2^	27.9 ± 3.6	28.0 ± 5.3	25.4 ± 2.9	25.3 ± 3.8
Fat percentage, %	30.1 ± 6.0	40.1 ± 7.0	25.8 ± 6.8	32.6 ± 5.7
Knee extension strength, kg	47.2 ± 10.0	30.2 ± 7.6	29.1 ± 9.4	22.2 ± 8.5
Knee extension strength (side of fracture), kg			20.2 ± 7.6	17.1 ± 7.3
Chair stand test, s	12.7 ± 2.6	14.8 ± 3.8	18.9 ± 3.6	23.1 ± 10.6
SPPB, score	10.6 ± 1.4	9.8 ± 1.5	5.9 ± 2.7	6.5 ± 2.2
Current self-rated health				
Very good/good	56 (45)	84 (45)	10 (56)	37 (63)
Average/poor	69 (55)	104 (55)	8 (44)	22 (37)
Smoking status				
Never smoker	56 (45)	135 (72)	8 (44)	52 (88)
Former smoker	60 (48)	48 (26)	7 (39)	3 (5)
Current smoker	9 (7)	5 (3)	3 (17)	4 (7)
Multimorbidity (≥ 2 diseases)	95 (76)	125 (67)	17 (94)	50 (85)
Musculoskeletal pain/disease	12 (10)	26 (14)	8 (44)	28 (48)
Cardiovascular disease	37 (30)	31 (17)	12 (67)	40 (68)
Pulmonary disease	12 (10)	27 (14)	1 (6)	14 (24)
Osteoarthritis	18 (14)	54 (29)	n/a	n/a
Diabetes	22 (18)	16 (9)	n/a	n/a
Stroke/Transient ischemic attack	7 (6)	10 (5)	n/a	n/a
Heart failure/valve diseases	9 (7)	7 (4)	n/a	n/a
Sarcopenia determinants				
ASMM, kg	23.8 ± 2.9	16.4 ± 2.0	22.6 ± 2.4	17.1 ± 2.3
ASMI, kg/m^2^	7.9 ± 0.8	6.4 ± 0.7	7.5 ± 0.6	6.9 ± 0.9
Grip strength, kg	37.4 ± 9.7	21.4 ± 5.8	29.0 ± 7.4	17.4 ± 6.8
Gait speed, m/s	1.3 ± 0.2	1.3 ± 0.2	0.9 ± 0.3	0.9 ± 0.2
Self-reported PA category				
0	18 (14)	24 (13)	3 (17)	7 (12)
1	34 (27)	49 (26)	6 (33)	8 (14)
2	20 (16)	52 (28)	4 (22)	42 (71)
3	33 (26)	42 (23)	4 (22)	0 (0)
4	19 (15)	20 (11)	0 (0)	1 (2)
5	1 (1)	0 (0)	1 (6)	0 (0)
Accelerometer-measured PA				
Valid days	6.7 ± 0.7	6.6 ± 0.8	n/a	n/a
Wear time, h/d	14.3 ± 1.3	13.9 ± 1.2	n/a	n/a
SED, min/d	625.3 ± 78.8	584.6 ± 79.9	n/a	n/a
LPA, min/d	197.6 ± 60.6	219.8 ± 68.6	n/a	n/a
MVPA, min/d	33.2 ± 21.1	32.1 ± 19.5	n/a	n/a

PA, physical activity; SPPB, short physical performance battery; ASMM, appendicular skeletal muscle mass; ASMI, appendicular skeletal muscle mass index; SED, sedentary physical activity; LPA, low physical activity; MVPA, moderate to vigorous physical activity

Values are presented as age-adjusted estimated marginal means with ± standard deviations or frequencies (%)

The prevalence of sarcopenia in older adults not meeting the PA guidelines was 3% (2% in women and 4% in men), while the prevalence of probable sarcopenia was 13% (16% in women and 8% in men) (Table 2). The prevalence of sarcopenia in the HF group was 3% (0% in women and 11% in men), while the prevalence of probable sarcopenia was 40% (41% in women and 39% in men). Only one man in the HF group met the criteria for severe sarcopenia.

**Table 2:** Prevalence of sarcopenia in older adults not meeting physical activity guidelines and older adults with recent hip fractures

	Women					Men				
		Probable sarcopenia		Sarcopenia			Probable sarcopenia		Sarcopenia	
	N	fr.	%	fr.	%	N	fr.	%	fr.	%
Not meeting PA guidelines, all	188	31	16.5	3	1.6	125	10	8.0	5	4.0
70–74 years old	117	12	10.3	2	1.7	74	5	6.8	3	4.1
75–79 years old	51	13	25.5	1	2.0	39	4	10.3	0	0.0
> 80 years old	20	6	30.0	0	0.0	12	1	8.3	2	16.7
Hip fracture, all	59	24	40.7	0	0.0	18	7	38.9	2	11.1
63–79 years old	26	6	23.1	0	0.0	8	3	37.5	1	12.5
> 80 years old	33	18	54.5	0	0.0	10	4	40.0	1	10.0

Fr, frequency; PA, physical activity

Differences between sarcopenia groups in baseline values were analyzed, and all age-adjusted estimated mean value differences between sarcopenia groups are presented in an additional file in more detail (see Additional file 1). In older adults not meeting PA recommendations, women in probable sarcopenia group had higher age (*p* < 0.05) and lower knee extension strength (*p* < 0.01), men in probable sarcopenia group had lower gait speed (*p* < 0.01), and men in sarcopenia group had lower knee extension strength (*p* < 0.001) and lower SPPB scores (*p* < 0.05) compared to the no sarcopenia group. In older adults with a recent HF, women in probable sarcopenia group had lower knee extension strength in fractured and non-fractured side (*p* < 0.05), and men in probable and confirmed sarcopenia groups had lower gait speed compared to the no sarcopenia group (*p* < 0.05). No differences in the self-reported amount of PA or accelerometer-measured PA in older adults not meeting the PA guidelines were found between the sarcopenia groups.

In the group of older adults not meeting the PA guidelines, those who reported having mainly casual walks and light outdoor recreation 1–2 times or several times per week (self-reported PA categories 1 and 2) were associated with higher risk (OR 5.3 95% CI 1.2–24.6, *p* < 0.05 and OR 5.5, 95% CI 1.2–25.7, *p* < 0.05, respectively) of probable sarcopenia and sarcopenia compared to those who reported having brisk physical activity 3–5 times per week or fitness exercises several times per week (category 4–5) (Table 3). No association with the risk of probable sarcopenia or sarcopenia was found in those reporting not moving more than necessary (category 0). In second model, combining those who reported having no more than necessary, or mainly casual walks and light recreation 1–2 times or several times per week (categories 0–2) were in a 2.8 times higher risk (95% CI 1.3–6.1, *p* = 0.009) of probable sarcopenia and sarcopenia compared to those who were having any brisk activities (categories 3–5). In addition, 1-year older age was associated with a 10% higher risk (OR 1.1, 95% CI 1.0–1.2, *p* = 0.007) of sarcopenia or probable sarcopenia. There were no associations between accelerometer-measured PA times and sarcopenia.

**Table 3:** Associations between probable and confirmed sarcopenia and self-reported and accelerometer-measured physical activity in older adults not meeting the physical activity guidelines (*n* = 313)

	No sarcopenia	Probable sarcopenia and sacopenia		
	OR	OR	95% CI	*p* value
Age (years)	1.0	1.1	1.0–1.2	0.007
Sex				
Women	1.0	reference		
Men	1.0	0.6	0.3–1.2	0.149
Category for self-reported PA^a^				0.083
0	1.0	3.2	0.6–17.3	0.171
1	1.0	5.3	1.2–24.6	0.032
2	1.0	5.5	1.2–25.7	0.031
3	1.0	2.2	0.4–11.4	0.340
4–5	1.0	reference		
Category for self-reported PA^a^				
0–2	1.0	2.8	1.3–6.1	0.009
3–5	1.0	Reference		
PA Model 1^b^				
SED time (30 min/day)	1.0	1.0	0.9–1.1	0.782
LPA time (30 min/day)	1.0	1.0	0.9–1.2	0.976
MVPA time (30 min/day)	1.0	0.8	0.4–1.3	0.304
PA Model 2^c^				
SED time (30 min/day)	1.0	1.0	0.8–1.1	0.530
LPA time (30 min/day)	1.0	1.0	0.9–1.2	0.971
MVPA time (30 min/day)	1.0	0.7	0.4–1.3	0.229

Reference: no sarcopenia. Sarcopenia refers to sarcopenia and probable sarcopenia

OR, odds ratio; CI, confidence interval; PA, physical activity

^a^ Adjusted for age and sex

^b^ Model 1 adjusted for age and sex. Only one activity variable was used as an exposure per regression

^c^ Model 2 further adjusted for other intensities of physical activity

Category 0 is for the lowest and 5 for the highest levels of self-reported PA. Categories 4 and 5 were combined due to the low response frequency in category 5. There were 21 participants missing data on accelerometer-measured PA (*n* = 21)

In the HF group, those who reported having mainly sitting activities (self-reported PA category 1) had a higher risk (OR 13.8, 95% CI, 1.1–173.0, *p* < 0.05) of sarcopenia than those who self-reported having moderate physical activity at least 3 h per week (PA categories 3 to 5) (Table 4). When categories were combined, those who reported mainly resting or sitting (categories 0 and 1) had a higher (OR 3.9, 95% CI, 1.4–11.3, *p* < 0.05) risk of having probable sarcopenia or sarcopenia than those with low to moderate physical activity (categories 2–5).

**Table 4:** Associations between probable and confirmed sarcopenia and self-reported physical activity in older adults with recent hip fractures (*n* = 77)

	No sarcopenia	Probable sarcopenia and sarcopenia		
	OR	OR	95% CI	*p* value
Age (years)	1.0	1.0	1.0–1.1	0.168
Sex				
Women	1.0	reference		
Men	1.0	1.5	0.5–4.2	0.485
Category for self-reported PA^a^				0.069
0	1.0	8.2	0.6–114.4	0.117
1	1.0	13.8	1.1–173.0	0.042
2	1.0	3.3	0.3–39.6	0.340
3–5	1.0	reference		
Category for self-reported PA^a^				
0–1	1.0	3.9	1.4–11.3	0.012
2–5	1.0	reference		
Category for self-reported PA^a^				0.032
0–1	1.0	11.2	1.0–127.0	0.050
2	1.0	3.5	0.3–40.8	0.965
3–5	1.0	reference		

Reference: no sarcopenia

OR, odds ratio; CI, confidence interval; PA, physical activity

^a^ Adjusted for age and sex

Category 0 was for the lowest and 5 for the highest levels of self-reported PA. Categories 4 and 5 were combined with category 3 due to low response frequencies in categories 4 and 5. Additional models with recategorized categories were used due to the low response frequencies in the lowest categories. One participant was missing data on self-reported physical activity

## Discussion

The prevalence of sarcopenia was 3% in both community-dwelling older adults not meeting the PA guidelines and in those with recent HF who were able and motivated to participate in exercise interventions. The prevalence of probable sarcopenia was 13% and 40%, respectively. A lower self-reported amount of PA was associated with an increased risk of sarcopenia in both study groups. However, no associations with accelerometer-measured PA levels and probable sarcopenia or sarcopenia were found in older adults not meeting the PA guidelines. This study provides evidence that a self-reported low level of physical activity is associated with sarcopenia in community-dwelling older adults who are not meeting recommended PA guidelines and those with recent HF.

This study provides information on sarcopenia in two populations that can be distinguished according to the discussed conceptual framework of acute and chronic sarcopenia [9, 16]. Participants not meeting the PA guidelines represent a risk group for developing chronic sarcopenia, and those with HF represent a risk group for developing acute sarcopenia. The low prevalence of sarcopenia (3%) in both study groups in this study might be explained by different factors. The exclusion criteria in both study groups excluded those with severe chronic diseases or conditions that effects on physical function. Only participants who were able to walk 500 m without assistance or walking aid in older adults not meeting the PA guidelines were included. All the participants were motivated to take part in exercise interventions, which might cause sampling bias. Additionally, in the HF group, 60% of participants self-rated their health as good or very good, indicating that the healthiest individuals might have participated in the original study. In addition, the use of bioimpedance analysis might have underestimated the prevalence of sarcopenia in those with recent hip fractures since it has been shown that bioimpedance overestimates muscle mass in older adults [49, 50].

When considering all above-mentioned factors, it is likely that the prevalence of sarcopenia is higher in unselected Finnish community-dwelling older adults with insufficient PA or HF. Actually, Patil et al. (2011) proposed the same, that the prevalence of sarcopenia in unselected Finnish population is likely higher when, in their study, the prevalence of sarcopenia according to EWGSOP 2010 was only 0.9% in Finnish women participating in vitamin D and exercise studies [51]. In comparison, in a recent meta-analysis, the prevalence of sarcopenia in community-dwelling European men and woman older than 65 years old (*n* = 4874) was 13–14% [11]. Thus, the low prevalence of sarcopenia in this study is a positive finding suggesting that older HF patients that are discharged to home and not institutionalized after surgery, and community-dwelling older adults who are not currently meeting the PA guidelines who are able and willing to participate in PA interventions are at a low risk for having sarcopenia.

In contrast, a high prevalence of probable sarcopenia, defined as low handgrip strength, was found in both study groups, especially in those with recent hip fractures (40%). An earlier systematic review found that lower grip strength was associated with the incidence of hip fracture in all 11 included cross-sectional and cohort studies [52]. Similarly, Harvey et al. found an association between low grip strength and hip fracture incidence in a meta-analysis of 5660 men with a mean follow-up time of 8.7 to 10.9 years [53]. In addition, hip fracture and surgery-related adverse outcomes, such as inflammatory reactions [22], immobilization, and bedrest, can lead to muscle wasting and decreased muscle strength [54]. Furthermore, the prevalence of sarcopenia has been found to increase 26–41% after a 1- to 2-year period after hip fracture [26]. Considering these findings, in addition to those of this study, the population of older adults with recent HF is at high risk for having probable sarcopenia and could develop acute sarcopenia in the near future after fractures.

Self-reported lower levels of PA were associated with a higher risk of sarcopenia in both study groups, while in older adults not meeting the PA guidelines, no associations between sarcopenia and accelerometer-measured PA were found. As earlier study shows, that correlation between self-reported PA and accelerometer measured PA levels are low (*r* = 0.105–0.318) [41], meaning that measurements measure slightly different things. Still, unexpected results that lower objectively measured PA levels are not associated with sarcopenia might be explained by those with sarcopenia or the lowest levels of PA perhaps increasing their PA during the accelerometer measurements, leading to dissipation of differences in PA levels between sarcopenia groups. As discussed earlier, measuring PA alone could improve PA habits and lead to improved levels of PA during measurements [55]. In addition, even if participants with normal levels of grip strength were performing any muscle strengthening activities, the hip-worn accelerometer does not count such activities well or might misinterpret them as light PA instead of MVPA. Another unexpected finding was that adults not meeting the PA guidelines who self-reported not moving more than necessary (category 0) had no significant association with higher sarcopenia risk. Although not within the scope of the present study, we explored this phenomenon and found that participants not meeting the PA guidelines in category 0 showed markedly higher body mass index (30.6 kg/m^2^) than participants who self-reported higher levels of PA (26.6–28.5 kg/m^2^). As a higher body mass index could have a protective role against sarcopenia [56], it might explain why participants with PA category 0 did not have a significantly higher sarcopenia risk.

In the HF group, adherence to PA was low, whereas 92% of participants reported mostly resting, sitting or low PA. The lowest levels (resting or sitting) of PA indicated a higher risk of sarcopenia. In earlier studies, it was shown that PA was associated with functional recovery after HF. Higher levels of PA predict better functional recovery [57], and remaining sedentary increases the risk for second HF and further functional decline [58]. It is also known that sarcopenia-related lower muscle mass, decreased amino acid reserve and release of amino acids could impair recovery from trauma [59, 60]. Also aging related muscles fat infiltration, myosteatosis, that is seen in sarcopenic individuals, is common among post-surgical patients and it increases metabolic and muscle dysfunctions [15]. These phenomena might explain some of the associations with sarcopenia and lower PA levels, as both impairs recovery after HF and might prolong sedentary behavior. Thus, multifactorial reasons for sarcopenia development [12] should be considered as the rationale of low PA levels after HF in sarcopenic individuals.

There are two important confounding factor that may affect the results for participants with HF. HF patients received standard care, which may have had temporal effects on muscle strength, mass, functional outcomes, and physical activity levels. After the hip fracture standard care included home-based exercise for lower limbs, only 50% of participants adhered to standard care after discharged to home, and of all participants 12 got referral to physiotherapy. In addition, as no general guidance for increasing the physical activity besides strengthening exercises were given, it is safe to say, that standard care or physiotherapy may not have had significant effect on baseline physical activity levels of participants with HF. Secondly, timeline for the baseline measurements in participants with HF ranged from approximately a month post-fracture to roughly 3 months post-fracture. This variation of recovery time may have confounded the activity levels and strength and function outcomes.

In comparison to this study, a meta-analysis conducted in 2017 including mostly studies with self-reported PA levels (24 of 25 studies) found that lower reported PA levels were associated with an up to 2-fold increase risk for sarcopenia [17]. Earlier studies exploring the associations between objectively recorded PA and sarcopenia have found that lower PA intensities are associated with a higher risk of sarcopenia [61–68]. Those studies showed that higher SED time [63, 64] and lower MVPA time [64–67] were associated with higher sarcopenia risk, while the amount of LPA had no associations with sarcopenia. In isotemporal substitution models, in which SED was substituted with MVPA, the risk of sarcopenia was shown to decrease, while substituting SED time with LPA had no similar effect [64, 66].

These associations may be explained by few different reasons. Sedentary behavior may displace higher intensities of PA [69]. Sedentary behavior is associated with higher levels of chronic low-grade inflammation [70], which is associated with a higher risk of sarcopenia [71]. As opposite, MVPA is known to have many positive effects on individuals’ health, and it may include activities that have sufficient stimuli for muscle strength maintenance. Association between LPA and sarcopenia is not so clear, and it is possible that low intensity activities do not generate enough stimuli to maintain muscle strength among older adults. To summarize these findings and the present study, it is important to minimize sedentary time and increase moderate-to-vigorous physical activity for reducing the risk of sarcopenia.

Physical activity and exercise play an important role preventing and treating sarcopenia, improving physical functioning, and preventing falls in older adults, all of which might affect health care costs. Therefore, it is important to study how to best promote physical activity and exercise in those, who are in a high risk for sarcopenia. In addition, studies on cost-efficiency of physical exercise on sarcopenia patients are needed to support health care decision-making concerning older adults in a high risk of sarcopenia.

### Strengths and limitations

The strengths of this study are carefully characterized populations with sufficient demographic data and different muscle strength, muscle mass, and functional outcomes, that helps to interpret findings and compare results to other studies or populations. Our study uses the most recent definition of sarcopenia, wherein muscle strength, muscle mass and physical performance were measured with recommended methods. Participants had an identified risk for sarcopenia but were in such a condition that lifestyle interventions could still be effective for preventing sarcopenia. In addition, our study was the first to explore the prevalence of sarcopenia with the latest definitions in the Finnish population.

The most important limitation that should be acknowledged is that baseline data were gathered from individuals who were going to participate in physical exercise intervention. This fact might have caused sampling bias, while those with the least physical activity or poor condition might not have been volunteering for the study and those with higher socioeconomic status might be over-represented in the study. As higher socioeconomic status is associated with lower sarcopenia and chronic disease risk [18], the results of this study might be too optimistic, when comparing results to whole population of older adults not meeting the PA guidelines or those with recent HF. In addition, the exclusion criteria for the PASSWORD study excluded those who were unable to walk 500 m without assistance, which may result in a study group of higher functioning older adults when compared to unselected population. This sampling bias is possibly seen in the characteristics of those not meeting PA guidelines as having higher average MVPA than expected on account of the study intake criteria. The body composition of older adults with recent hip fractures was measured with bioimpedance, which is not as reliable and valid for measuring muscle mass as DXA. Duration of accelerometer measurements were relatively short to describe long-term PA habits. In addition, 7% of participants not meeting the PA guidelines were missing accelerometer measurement data. Because of the small number of individuals with sarcopenia, it is important to interpret the associations and risks of sarcopenia with caution. As populations in this study were relatively small and had selection bias, hypothesis generating result is deserved to be investigated in larger study samples.

## Conclusion

In conclusion, the prevalence of sarcopenia in community-dwelling older adults not meeting the PA guidelines and in those with recent HF who can participate in PA intervention is low. However, the prevalence of probable sarcopenia in these populations, especially in those with recent HF, is high. Therefore, we recommend screening for low muscle strength and promoting PA in those with any signs of sarcopenia to delay and prevent sarcopenia. In addition, simple questions clarifying the amount and intensity of daily habitual PA could provide valuable information when assessing the risk of developing sarcopenia in older adults.

### Electronic supplementary material

Below is the link to the electronic supplementary material.

Supplementary Material 1

## Data Availability

Datasets used during the current study are available from the author (SS) on reasonable request and are possible after the complete data collection has been finalized, and the datasets have been anonymized.

## Abbreviations

ASMI: Appendicular skeletal muscle mass index
ASMM: Appendicular skeletal muscle mass
CI: Confidence interval
DXA: Dual-energy X-ray absorptiometry
EWGSOP: European Working Group in Older People
FP: Fat percent
HF: Hip fracture
LPA: Light physical activity
MMSE: Mini Mental State Examination
MVPA: Moderate to vigorous physical activity
OR: Odds ratio
PA: Physical activity
PASSWORD: The Promoting Safe Walking Among Older People study
ProMo: The Promoting Mobility After Hip Fracture study
RCT: Randomized controlled trial
SD: Standard deviation
SED: Sedentary behaviour
SPPB: Short Physical Performance Battery
