# Supplementary material for: Low physical activity is a risk factor for sarcopenia: a cross-sectional analysis of two exercise trials on community-dwelling older adults
Source: BMC Geriatr. 2024 Feb 29;24:212. doi: 10.1186/s12877-024-04764-1 (PMC10905947; doi:10.1186/s12877-024-04764-1)
Supplement: Supplementary file 1 — Supplementary Material 1 [file 12877_2024_4764_MOESM1_ESM.docx]

Supplementary data. Age adjusted characteristics according to sarcopenia status in women and men not meeting the physical activity guidelines.

|  | | Women | | | | Men | | | |
| --- | --- | --- | --- | --- | --- | --- | --- | --- | --- |
|  | | No sarcopenia | Probable sarcopenia | Sarcopenia | p value^a^ | No sarcopenia | Probable sarcopenia | Sarcopenia | p value^a^ |
| n | | 154 | 31 | 3 |  | 110 | 10 | 5 |  |
| Age, years | | 74.2 ± 3.7 | 76.3 ± 3.9* | 74.4 ± 3.8 | 0.018 | 74.3 ± 3.8 | 75.6 ± 6.3 | 75.8 ± 6.3 | 0.624 |
| Body mass index, kg/m^2^ | | 27.9 ± 0.4 | 28.8 ± 1.0 | 23.0 ± 3.1 | 0.195 | 27.9 ± 0.3 | 29.1 ± 1.1 | 25.4 ± 1.6 | 0.156 |
| Fat percent, % | | 39.9 ± 0.6 | 41.9 ± 1.3 | 37.5 ± 4.0 | 0.290 | 30.0 ± 0.6 | 30.4 ± 26.6 | 32.5 ± 2.7 | 0.672 |
| Knee extension strength, kg | | 31.1 ± 0.6 | 26.3 ± 1.2** | 25.5 ± 3.9 | <0.001 | 48.4 ± 0.8 | 41.8 ± 2.8 | 33.2 ± 3.9*** | <0.001 |
| Chair stand test, s | | 14.5 ± 0.3 | 15.9 ± 0.7 | 16.6 ± 2.1 | 0.147 | 12.5 ± 0.2 | 12.9 ± 0.8 | 15.3 ± 1.2 | 0.076 |
| SPPB, score | | 9.9 ± 0.1 | 9.4 ± 0.3 | 10.0 ± 0.9 | 0.181 | 10.7 ± 0.1 | 10.1 ± 0.4 | 8.8 ± 0.6* | 0.006 |
| Sarcopenia determinants | |  |  |  |  |  |  |  |  |
| Grip strength, kg | | 23.1 ± 0.4 | 13.5 ± 0.8** | 14.6 ± 2.5*** | <0.001 | 39.4 ± 0.7 | 22.6 ± 2.3*** | 22.9 ± 3.3*** | <0.001 |
| ASMM, kg | | 16.5 ± 0.2 | 16.1 ± 0.4 | 13.3 ± 1.1* | <0.001 | 24.1 ± 0.2 | 23.2 ± 0.8 | 18.4 ± 1.2*** | <0.001 |
| ASMI, kg | | 6.4 ± 0.1 | 6.3 ± 0.1 | 5.2 ± 0.4* | 0.014 | 7.9 ± 0.1 | 8.0 ± 0.2 | 6.2 ± 0.3*** | <0.001 |
| Gait speed, m/s | | 1.3 ± 0.0 | 1.3 ± 0.0 | 1.3 ±0.1 | 0.576 | 1.3 ± 0.0 | 1.2 ± 0.1** | 1.2 ± 0.1 | <0.001 |
| Physical activity | |  |  |  |  |  |  |  |  |
| SED (min/d) | | 582.7 ± 6.8 | 593.5 ± 15.5 | 595.0 ± 46.5 | 0.796 | 629.0 ± 7.6 | 599.8 ± 24.6 | 598.9 ± 34.8 | 0.394 |
| LPA (min/d) | | 220.2 ± 5.8 | 216.8 ± 12.1 | 231.1 ± 39.3 | 0.934 | 197.0 ± 5.8 | 196.1 ± 19.0 | 213.7 ± 26.8 | 0.829 |
| MVPA (min/d) | | 33.2 ± 1.6 | 25.9 ± 3.7 | 38.1 ± 11.0 | 0.168 | 32.9 ± 2.0 | 39.5 ± 6.5 | 25.4 ± 9.2 | 0.433 |
| Self-reported PA | |  |  |  | 0.076 |  |  |  | 0.319 |
| 0 | 19 (12) | 4 (13) | 1 (33) |  | 17 (15) | 1 (10) | 0 (0) |  |  |
| 1 | 34 (22) | 14 (45) | 1 (33) |  | 31 (28) | 2 (20) | 1 (20) |  |  |
| 2 | 42 (27) | 10 (32) | 0 (0) |  | 14 (13) | 4 (40) | 2 (40) |  |  |
| 3 | 40 (26) | 2 (6) | 1 (33) |  | 29 (26) | 3 (30) | 1 (20) |  |  |
| 4/5 | 19 (12) | 1 (3) | 0 (0) |  | 19 (17) | 0 (0) | 1 (20) |  |  |

ASMM, appendicular skeletal muscle mass; ASMI, appendicular skeletal muscle mass index; SPPB, short physical performance battery; SED, sedentary behavior; LPA, light physical activity; MVPA, moderate to vigorous physical activity.

P value^a^ is for age adjusted analysis of variance. Values are presented as age adjusted estimated marginal means with ± standard deviations or frequencies (%). Markers showing groups with statistically significant difference related to no sarcopenia group (post-hoc; Bonferroni *p<0.05, **p<0.005, ***p<0.001).

The number of participants with missing data for DXA variables (fat percent, ASMM, ASMI), knee extension strength, and accelerometer measured variables (SED, LPA, MVPA) were 1 man, 3 (1 man), and 21 (4 men), respectively.

Supplementary data. Age adjusted characteristics according to sarcopenia status in women and men recovering from a hip fracture.

|  | Women | | | Men | | | |
| --- | --- | --- | --- | --- | --- | --- | --- |
|  | No sarcopenia | Probable sarcopenia | P-value^a^ | No sarcopenia | Probable sarcopenia | Sarcopenia | P-value^a^ |
| n | 35 | 24 |  | 9 | 7 | 2 |  |
| Age, years | 78.0 ± 6.9 | 81.6 ± 7.8 | 0.067 | 80.6 ± 6.1 | 78.4 ± 5.0 | 79.0 ± 9.9 | 0.778 |
| Body mass index, kg/m^2^ | 25.6 ± 0.6 | 24.9 ± 0.8 | 0.489 | 25.4 ± 0.9 | 26.4 ± 1.1 | 22.3 ±1.9 | 0.208 |
| Fat percent, % | 32.4 ± 0.9 | 33.0 ± 1.2 | 0.683 | 24.2 ± 2.5 | 29.3 ± 2.8 | 21.8 ± 4.9 | 0.304 |
| Knee extension strength, kg | 24.2 ± 1.4 | 19.5 ± 1.7* | 0.04 | 30.5 ± 3.3 | 27.3 ± 3.8 | 28.6 ±7.0 | 0.822 |
| Knee extension strength fract., kg | 18.8 ± 1.2 | 14.6 ± 1.5* | 0.028 | 23.7 ± 2.4 | 16.9 ± 2.7 | 16.1 ± 5.0 | 0.154 |
| Chair stand test, s | 23.0 ± 2.0 | 23.4 ± 2.8 | 0.911 | 17.3 ± 1.3 | 20.4 ± 1.9 | 25.6 ± 3.6 | 0.139 |
| SPPB, scores | 6.9 ± 0.4 | 5.9 ± 0.5 | 0.093 | 6.9 ± 0.9 | 4.6 ± 1.0 | 5.9 ± 1.8 | 0.275 |
| Sarcopenia determinants |  |  |  |  |  |  |  |
| Grip strength, kg | 21.3 ± 0.7 | 11.8 ± 0.9*** | <0.001 | 34.8 ± 1.7 | 23.2 ± 1.9** | 23.8 ± 3.6* | 0.001 |
| ASMM, kg | 17.5 ± 0.4 | 16.4 ± 0.5 | 0.093 | 23.4 ± 0.8 | 22.4 ± 1.0 | 19.7 ± 1.6 | 0.172 |
| ASMI, kg | 6.9 ± 0.2 | 6.8 ± 0.2 | 0.708 | 7.7 ± 0.2 | 7.5 ± 0.2 | 6.7 ± 0.4 | 0.079 |
| Gait speed, m/s | 0.9 ± 0.04 | 0.8 ± 0.05 | 0.057 | 1.0 ± 0.06 | 0.7 ± 0.07* | 0.5 ± 0.1* | 0.003 |
| Self-reported physical activity |  |  | 0.116 |  |  |  | 0.303 |
| 0 | 2 (6) | 5 (21) |  | 2 (22) | 1 (14) | 0 (0) |  |
| 1 | 3 (9) | 5 (21) |  | 1 (11) | 3 (43) | 2 (100) |  |
| 2 | 28 (82) | 14 (58) |  | 2 (22) | 2 (29) | 0 (0) |  |
| 3-5 | 1 (3) | 0 |  | 4 (44) | 1 (14) | 0 (0) |  |

Fract., fractured side; ASMM, Appendicular skeletal muscle mass; ASMI, appendicular skeletal muscle mass index; SPPB, short physical performance battery

P value^a^ is for age adjusted variance analysis. Values are presented as age adjusted estimated marginal means with ± standard deviations or frequencies (%). Markers showing groups with statistically significant difference related to no sarcopenia group (post-hoc; Bonferroni *p<0.05, **p<0.005, ***p<0.001).

The number of participants with missing data for body mass index, bioimpedance values (Fat percent, ASMM, ASMI), non-fr. knee extension strength, fr. knee extension strength, and gait speed were 1 man, 5 (1 men), 1 woman, 5 women, and 1 woman, respectively. For chair stand test 22 participants were not able to do the test.
